# Supplementary material for: Zebrafish sleep displays distinct sub-states
Source: bioRxiv. 2025 Sep 2:2025.08.28.672887. Preprint. [Version 1] doi: 10.1101/2025.08.28.672887 (PMC12424785; doi:10.1101/2025.08.28.672887)
Supplement: Supplement 1 [file NIHPP2025.08.28.672887v1-supplement-1.pdf]

bioRxiv preprint doi: https://doi.org/10.1101/2025.08.28.672887; this version posted September 2, 2025. The copyright holder for this preprint (which was not certified by peer review) is the author/funder, who has granted bioRxiv a license to display the preprint in perpetuity. It is made available under aCC-BY 4.0 International license.

Supplementary Table 1. λ values for fish with 4 states as optimal

	λ_{S2}	λ_{S1}	λ_{W1}	λ_{W2}
fish 1	0.02	1.27	4.65	7.04
fish 2	0.01	0.23	0.91	5.89
fish 3	0.01	1.10	5.76	8.23
fish 7	0.01	1.33	3.67	8.97
fish 9	0.01	1.00	4.97	8.86
fish 10	0.00	1.27	4.18	5.87
fish 11	0.02	1.16	3.85	7.08
fish 18	0.02	0.94	3.17	8.52
fish 19	0.02	1.35	5.72	8.70

Supplementary Table 2. λ values for fish with 3 states as optimal

<table>
<tr><td>fish 4 (3 state)</td><td colspan="4">0.02</td><td colspan="4">1.55</td><td colspan="4">7.00</td></tr>
<tr><td>fish 4 (4 state)</td><td colspan="3">0.02</td><td colspan="3">1.29</td><td colspan="3">5.50</td><td colspan="3">8.65</td></tr>
<tr><td>fish 15 (3 state)</td><td colspan="4">0.03</td><td colspan="4">1.79</td><td colspan="4">8.91</td></tr>
<tr><td>fish 15 (4 state)</td><td colspan="3">0.03</td><td colspan="3">1.26</td><td colspan="3">2.23</td><td colspan="3">9.06</td></tr>
<tr><td>fish 17 (3 state)</td><td colspan="4">0.01</td><td colspan="4">2.15</td><td colspan="4">7.24</td></tr>
<tr><td>fish 17 (4 state)</td><td colspan="3">0.00</td><td colspan="3">1.21</td><td colspan="3">4.01</td><td colspan="3">7.91</td></tr>
<tr><td>fish 20 (3 state)</td><td colspan="4">0.01</td><td colspan="4">1.52</td><td colspan="4">4.77</td></tr>
<tr><td>fish 20 (4 state)</td><td colspan="3">0.00</td><td colspan="3">1.06</td><td colspan="3">3.41</td><td colspan="3">5.87</td></tr>
</table>

Supplementary Table 3. λ values for fish with 5 states as optimal

<table>
<tr><td>fish 5 (5 state)</td><td colspan="4">0.04</td><td colspan="4">1.11</td><td colspan="4">1.16</td><td colspan="4">4.62</td><td colspan="4">6.79</td></tr>
<tr><td>fish 5 (4 state)</td><td colspan="5">0.04</td><td colspan="5">1.16</td><td colspan="5">1.36</td><td colspan="5">5.33</td></tr>
<tr><td>fish 6 (5 state)</td><td colspan="4">0.04</td><td colspan="4">0.09</td><td colspan="4">1.28</td><td colspan="4">3.71</td><td colspan="4">5.12</td></tr>
<tr><td>fish 6 (4 state)</td><td colspan="5">0.05</td><td colspan="5">1.49</td><td colspan="5">3.92</td><td colspan="5">5.13</td></tr>
<tr><td>fish 8 (5 state)</td><td colspan="4">0.01</td><td colspan="4">0.11</td><td colspan="4">1.53</td><td colspan="4">3.14</td><td colspan="4">5.30</td></tr>
<tr><td>fish 8 (4 state)</td><td colspan="5">0.01</td><td colspan="5">0.31</td><td colspan="5">1.54</td><td colspan="5">4.28</td></tr>
<tr><td>fish 12 (5 state)</td><td colspan="4">0.03</td><td colspan="4">0.40</td><td colspan="4">1.23</td><td colspan="4">4.08</td><td colspan="4">7.69</td></tr>
<tr><td>fish 12 (4 state)</td><td colspan="5">0.03</td><td colspan="5">1.23</td><td colspan="5">2.49</td><td colspan="5">6.97</td></tr>
<tr><td>fish 13 (5 state)</td><td colspan="4">0.02</td><td colspan="4">0.18</td><td colspan="4">2.11</td><td colspan="4">3.45</td><td colspan="4">7.62</td></tr>
<tr><td>fish 13 (4 state)</td><td colspan="5">0.04</td><td colspan="5">2.11</td><td colspan="5">6.35</td><td colspan="5">9.56</td></tr>
<tr><td>fish 14 (5 state)</td><td colspan="4">0.01</td><td colspan="4">0.02</td><td colspan="4">1.29</td><td colspan="4">3.42</td><td colspan="4">7.73</td></tr>
<tr><td>fish 14 (4 state)</td><td colspan="5">0.00</td><td colspan="5">1.36</td><td colspan="5">4.93</td><td colspan="5">8.22</td></tr>
<tr><td>fish 16 (5 state)</td><td colspan="4">0.03</td><td colspan="4">0.70</td><td colspan="4">1.54</td><td colspan="4">4.62</td><td colspan="4">10.45</td></tr>
<tr><td>fish 16 (4 state)</td><td colspan="5">0.05</td><td colspan="5">1.50</td><td colspan="5">5.27</td><td colspan="5">10.63</td></tr>
</table>

bioRxiv preprint doi: https://doi.org/10.1101/2025.08.28.672887; this version posted September 2, 2025. The copyright holder for this preprint (which was not certified by peer review) is the author/funder, who has granted bioRxiv a license to display the preprint in perpetuity. It is made available under aCC-BY 4.0 International license.

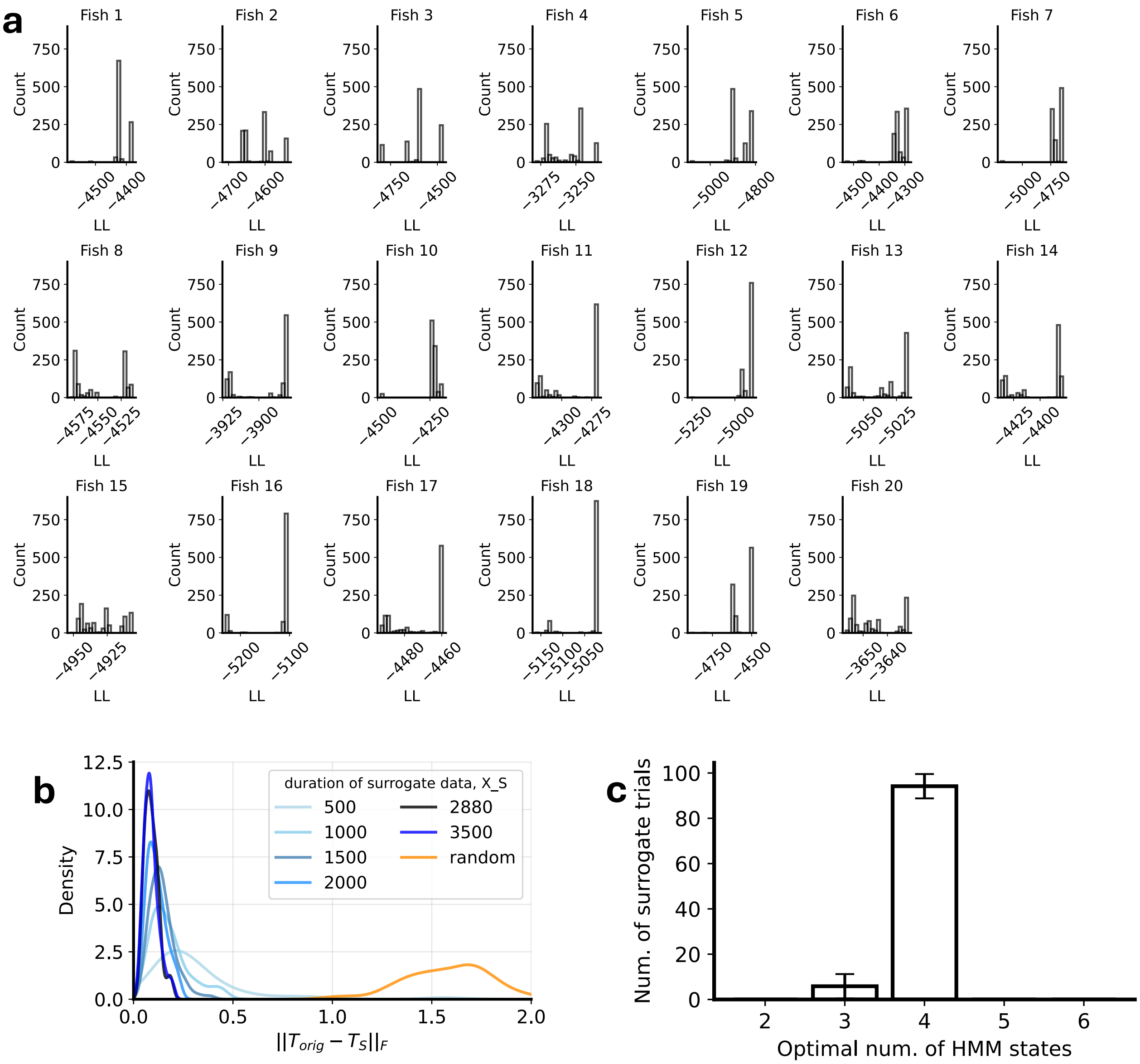

Figure S1: **Robustness of HMM parameter fits.** (**a**) Distribution of log-likelihood (LL) over 1000 model fits for each WT fish, depicting variability among different runs. (**b**) Distribution of difference (Frobenius norm) between the transition probability matrix T_{orig} fitted on 48 h fish data and the transition probability matrix fitted on surrogate data generated from T_{orig}, as a function of the amount of surrogate data provided. The distributions became narrower as longer durations of data were used for fitting, with little change after duration of 2880 minutes. For the random distribution case each row of the transition probability matrix was generated from a Dirichlet distribution and normalized to sum to one. (**c**) For surrogate data generated 100 times from a 4-HMM fit to a fish for which 4 states was optimal, the optimal number of fitted states was 4 in 94 cases. The error bars are standard error of mean over fish.

bioRxiv preprint doi: https://doi.org/10.1101/2025.08.28.672887; this version posted September 2, 2025. The copyright holder for this preprint (which was not certified by peer review) is the author/funder, who has granted bioRxiv a license to display the preprint in perpetuity. It is made available under aCC-BY 4.0 International license.

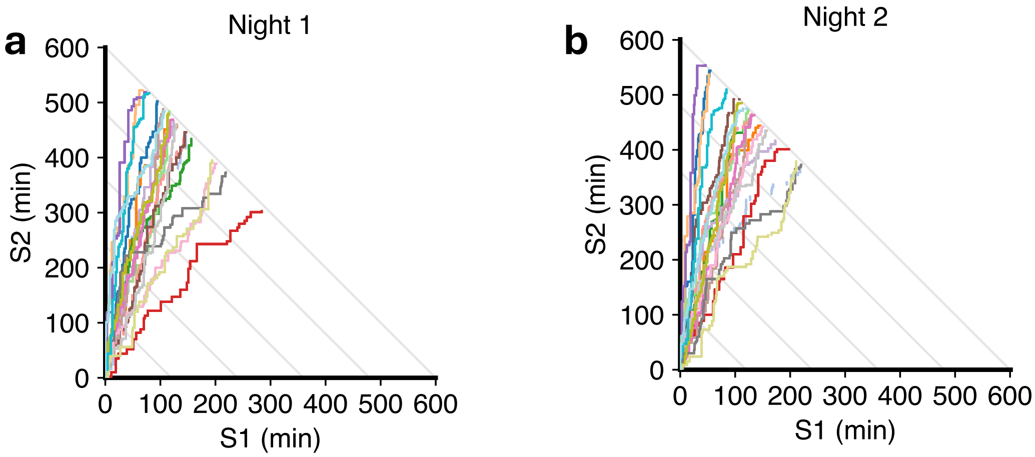

Figure S2: **Complete S1 and S2 state sequences at night for all WT fish.** In this representation night starts at the origin, and then we draw a horizontal line for each minute in state S1 and a vertical line for each minute in S2. Each color corresponds to a different fish (matched between nights). In the rare instances when a wake state occurs we leave a horizontal space. All lines thus finish at 600 total min (10 h). These diagrams provide a compact way of representing individual sleep-state sequences for all fish in the same plot.

bioRxiv preprint doi: https://doi.org/10.1101/2025.08.28.672887; this version posted September 2, 2025. The copyright holder for this preprint (which was not certified by peer review) is the author/funder, who has granted bioRxiv a license to display the preprint in perpetuity. It is made available under aCC-BY 4.0 International license.

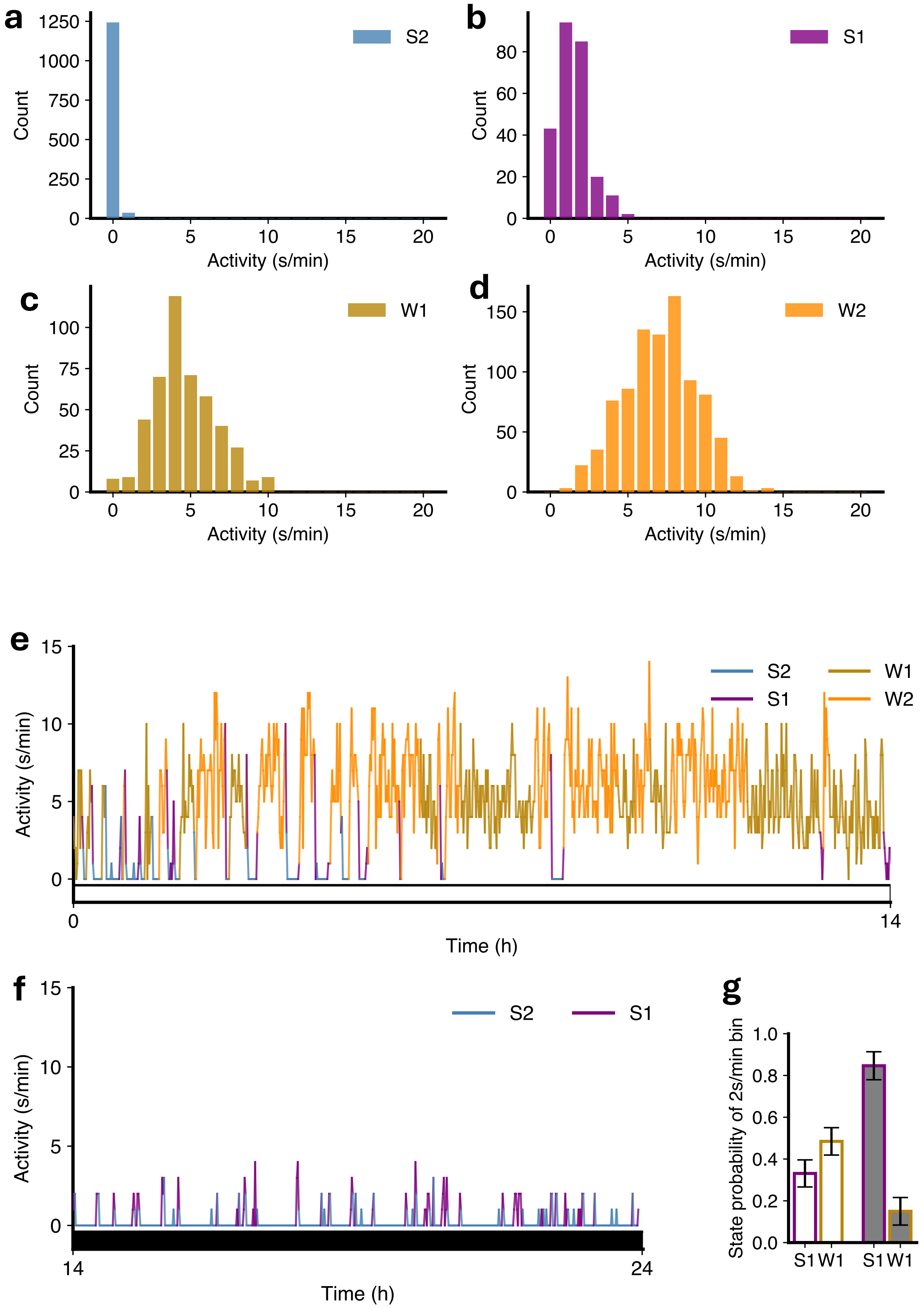

Figure S3: **Relation of activity levels to state assignments and arousal probability.** (**a-d**) Histograms for an example WT fish showing the distribution of activity levels assigned to each of the four states. (**e-f**) Activity levels of the same fish colored by the state assigned at each time during the first day (**e**) and night (**f**). (**g**) On average, bins with 2 s/min of activity are more likely to get assigned state W1 during the day (empty bars), but state S1 during the night (filled bars).

bioRxiv preprint doi: https://doi.org/10.1101/2025.08.28.672887; this version posted September 2, 2025. The copyright holder for this preprint (which was not certified by peer review) is the author/funder, who has granted bioRxiv a license to display the preprint in perpetuity. It is made available under aCC-BY 4.0 International license.

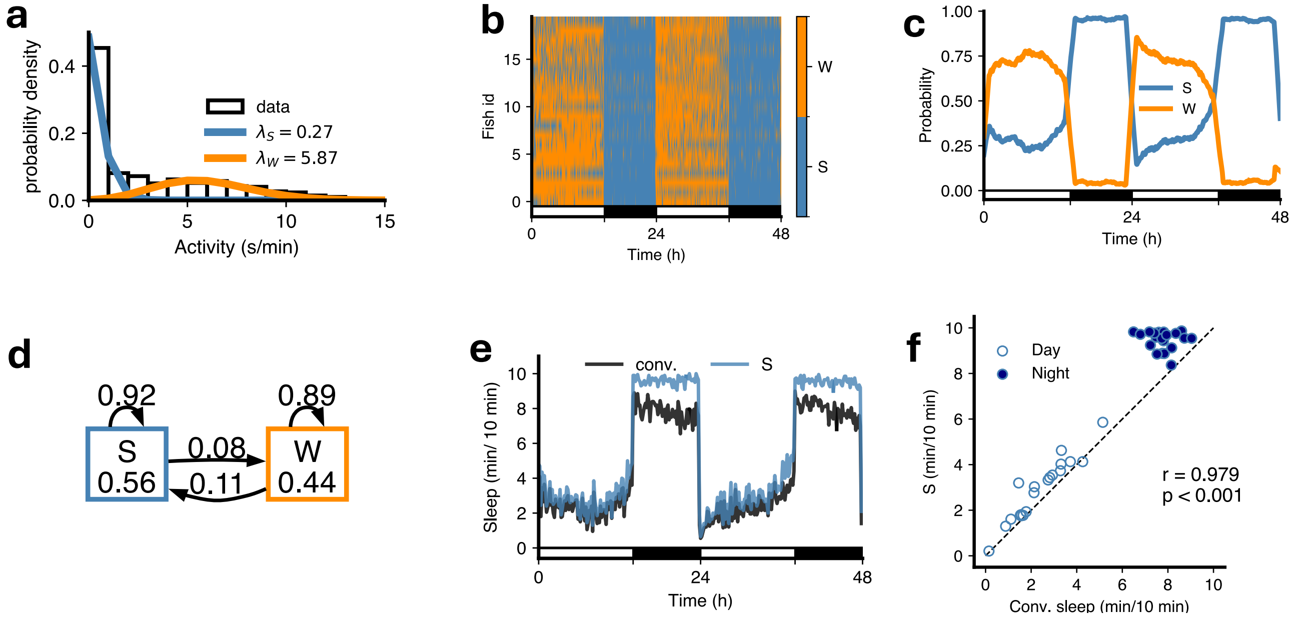

Figure S4: **A 2-HMM fit to WT fish data.** (**a**) λs for Poisson distribution fit to activities in both states (S and W). (**b**) Most likely state sequences underlying fish activities. (**c**) State probabilities during day and night phases. (**d**) Transition diagram for the model. (**e**) A comparison of sleep amounts for the S state and sleep calculated conventionally. (**f**) HMM sleep versus conventional sleep for each fish during day and night. r and p-value are for Pearson correlation for the fit to the line y=x.

bioRxiv preprint doi: https://doi.org/10.1101/2025.08.28.672887; this version posted September 2, 2025. The copyright holder for this preprint (which was not certified by peer review) is the author/funder, who has granted bioRxiv a license to display the preprint in perpetuity. It is made available under aCC-BY 4.0 International license.

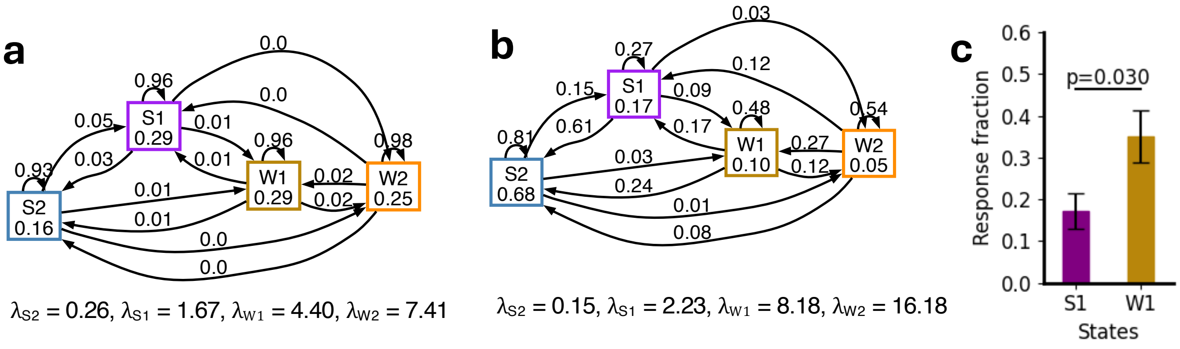

Figure S5: **HMM parameters for sleep deprivation and arousal experiments.** (**a**) Mean transition diagram and λ values for the HMM fit to sleep deprivation data. (**b**) Mean transition diagram and λ values for the HMM fit to arousal experiment data. (**c**) Response fractions from state S1 versus state W1, both corresponding to activity level of 5 s/min before the stimulus.

bioRxiv preprint doi: https://doi.org/10.1101/2025.08.28.672887; this version posted September 2, 2025. The copyright holder for this preprint (which was not certified by peer review) is the author/funder, who has granted bioRxiv a license to display the preprint in perpetuity. It is made available under aCC-BY 4.0 International license.

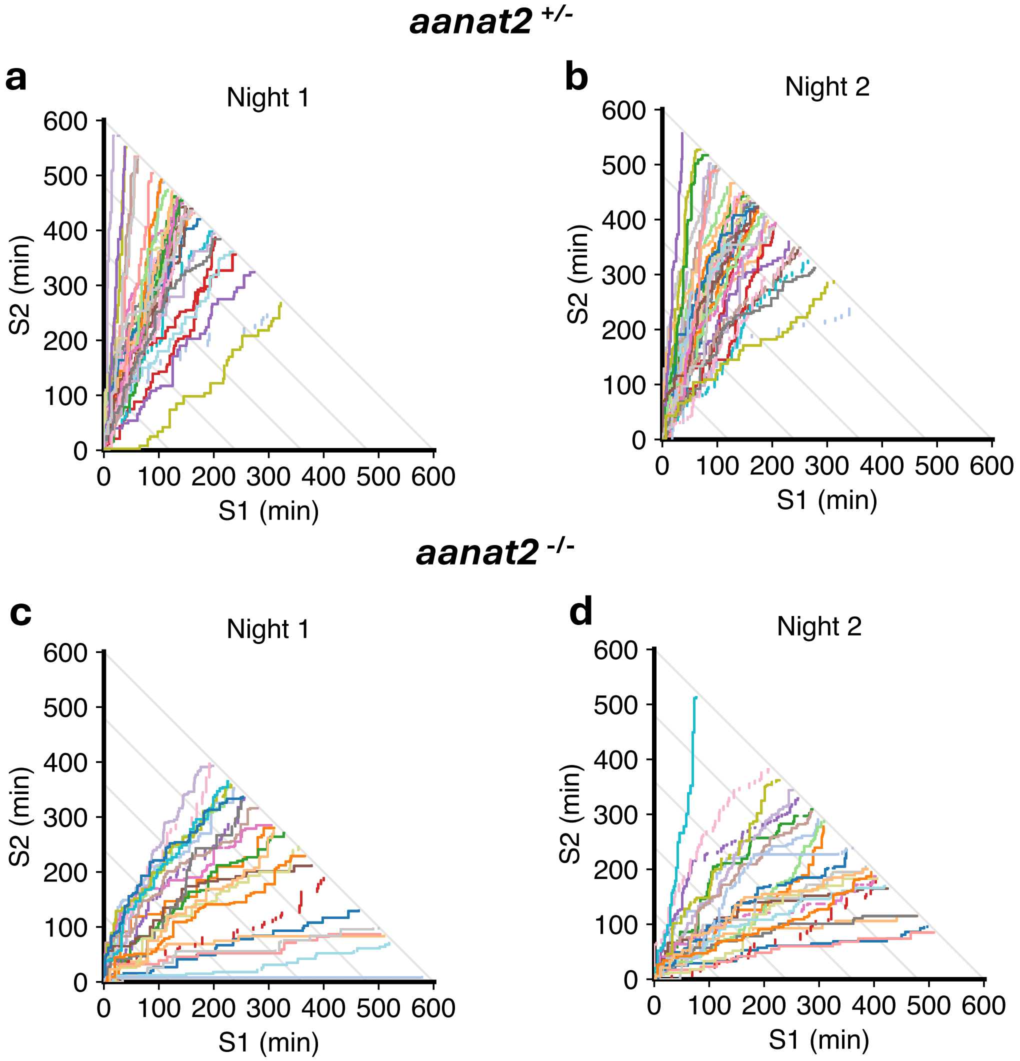

Figure S6: **Comparison of S1 and S2 state sequences during night for *aanat2*$^{+/-}$ and *aanat2*$^{-/-}$ fish.** (**a-b**) *aanat2*$^{+/-}$ fish. (**c-d**) *aanat2*$^{-/-}$ fish. Colors represent different fish. The increase in S1 relative to S2 in *aanat2*$^{-/-}$ fish is clearly apparent.
